# Alexithymia is associated with submissive behavior in a public goods game

**DOI:** 10.1192/j.eurpsy.2025.1930

**Published:** 2025-08-26

**Authors:** A. Carvallo, J. Gonzalez, V. V. Orozco, J. Hanna, J. A. Fernandez, J. Ayala, A. Barros, M. F. Aguirre, K. Arroyo, P. Campodonico, F. Munoz-Rubke, M. E. Riveros

**Affiliations:** 1 Facultad de Medicina CAS, Universidad del Desarrollo; 2 Facultad de odontología y Ciencias de la Rehabilitación, Universidad San Sebastian; 3 Centro de Química Médica, Universidad del Desarrollo; 4 Facultad de Medicina UDD (FM, UDD), Instituto de Inovacion en Medicina (ICIM), Santiago; 5 Instituto de Psicología, Universidad Austral de Chile, Puerto Montt; 6 Centro de Fisiología celular e Integrativa, Universidad del Desarrollo; 7 Facultad de Medicina (FM, UDD), Instituto de Inovacion en Medicina (ICIM), Santiago, Chile

## Abstract

**Introduction:**

Alexithymia is a relatively stable personality construct that reflects the difficulty in distinguishing and describing one’s own emotions along with a concrete and externally directed style of thinking. Alexithymia increases the risk of development and worsens the course of several psychiatric illnesses, such as depression or addiction. It is also associated with the development of eating disorders and psychosomatic illnesses. Additionally, it has a negative impact on the ability to regulate emotions and is also associated with a reduced empathic capacity, interpersonal problems and even violent behaviors. Interestingly, in intimate partner violence, it has been observed that alexithymia is associated with both exercising and receiving abuse. However, social behavior, in dynamics of cooperation within pairs and groups of several individuals, has not been studied much in relation to alexithymia.

**Objectives:**

We evaluated the impact of alexithymia in social behavior in three aspects: generosity, trust and submission.

**Methods:**

After completing an online survey that evaluated their levels of alexithymia using the TAS-20, as well as depression and loneliness, 67 participants (27 men), aged 19 to 46, attended our laboratory. There, after answering the PANAS survey, they played three economic games using tiles: the Dictator game, the Trust game, and the Public Goods game. Each participant played in a group of four, with three of the group members being confederates whose contributions were pre-established and consistent across all sessions and participants. In the Public Goods game, confederates initially contributed a significant percentage (80-95%) of their endowment in the first round, but in subsequent rounds, they drastically reduced their contributions to nearly nonexistent levels. After playing all three games, the PANAS was reassessed. Finally, on the same day as the face-to-face session, participants completed a second online survey that assessed cognitive and emotional empathy, early life adversity, resilience and perceived stress.

**Results:**

In the Public Goods game, the number of rounds in which participants contributed more than their initial contribution in the first round was used as a measure of submissive behavior. This variable correlated with the participants’ level of alexithymia (β=0.544). Additionally, this same index was also negatively associated with empathy and positively related to reported loneliness. A similar pattern was observed between alexithymia and empathy (β=-0.323) and loneliness (β=0.473). In contrast, total contributions made in the Public Goods game, the Dictator game (generosity), and the Trust game did not correlate with alexithymia.

**Conclusions:**

Our results suggest that alexithymia is connected to greater submissive behavior in group interactions. It is possible that its link to reduced empathy and increased loneliness contributes to this behavior.

**Disclosure of Interest:**

None Declared

